# Sildenafil-Induced Acute Fulminant Hepatic Failure: Tragedy After Triumph

**DOI:** 10.7759/cureus.27378

**Published:** 2022-07-27

**Authors:** Twinkle Pawar, Keyur Saboo, Mansi Patel, Sunil Kumar, Sourya Acharya

**Affiliations:** 1 Department of Medicine, Jawaharlal Nehru Medical College, Datta Meghe Institute of Medical Sciences, Wardha, IND

**Keywords:** encephalopathy, fulminant, phosphodiesterase, sildenafil, liver toxicity

## Abstract

Sildenafil citrate is a specific phosphodiesterase type 5-enzyme blocker that can enhance the indirect impacts of nitric oxide on vascular smooth muscle and permeability via the guanosine monophosphate (c-GMP) route in the penis erectile tissue. Though the medication is well taken, particular side effects such as flushes, headache, indigestion, and retinal abnormalities have indeed been reported. Liver toxicity caused by sildenafil use is thought to be quite infrequent. There have been few studies that looked at a potential link between sildenafil usage and liver problems, and the underlying cause involved for liver toxicity is still unknown. We report a unique instance of acute severe hepatitis with fulminant hepatic failure in a 38-year-old male patient after taking sildenafil citrate.

## Introduction

Sildenafil (a type 5 phosphodiesterase (PDE5) blocker) acts on PDE5, which is present mostly in the corpora cavernous of the penis and lung. PDE5 suppression prolongs the stimulation of cyclic guanosine monophosphate (cGMP), a significant modulator of nitric oxide's vasodilating effect. Phosphodiesterase inhibition prolongs sympathetic stimulation in the corpora cavernous of the penis (enhancing erections) and pulmonary hypertension (reducing pulmonary artery pressure). The suggested dose is 50 mg as a single injection one hour prior to sexual intercourse, with doses increasing or decreasing dependent on tolerance and efficacy, with a maximum rate of once a day and resources and capacities of 100 mg [1]. Fainting, headaches, flushes, hypotension, sneezing, and indigestion are common adverse effects. Sight and hearing impairment, hypotension, cardiovascular problems, and pelvic pain are all rare but life-threatening side effects [1].

There have been a few cases of acute liver damage associated with sildenafil usage, but no cases of severe liver failure. Due to the obvious inconsistent and even sometimes unrecognized use of sildenafil, the delay in most studies has indeed been unclear, but it seems to be between one to eight weeks. The distribution of serum enzyme increases, which shifts from hepatocytes to cholestatic, occasionally switching back and forth. The most compelling examples have involved moderate cholestatic or "mixed" hepatitis that developed within one to three months of commencing sildenafil. Autoantibodies and immunoallergic characteristics were not found. Cases of severe onset with elevated blood aminotransferase levels following sildenafil usage have been recorded, with some hallmarks of ischemia damage [2]. 

## Case presentation

A 38-year-old man presented with complaints of yellow discolouration of sclera and urine, altered sensorium, and irrelevant talk for two days. There has been no evidence of hematemesis, melena, stomach discomfort, vomiting, or distension. The patient denied any history of hypertension, diabetes, bronchial asthma, or tuberculosis. Two days earlier, he has taken sildenafil in 50 mg dose for sexual desire fulfilment, on the advice of a local private practitioner. No additional drugs were taken and he denied any history of alcohol consumption and smoking.

**Figure 1:**
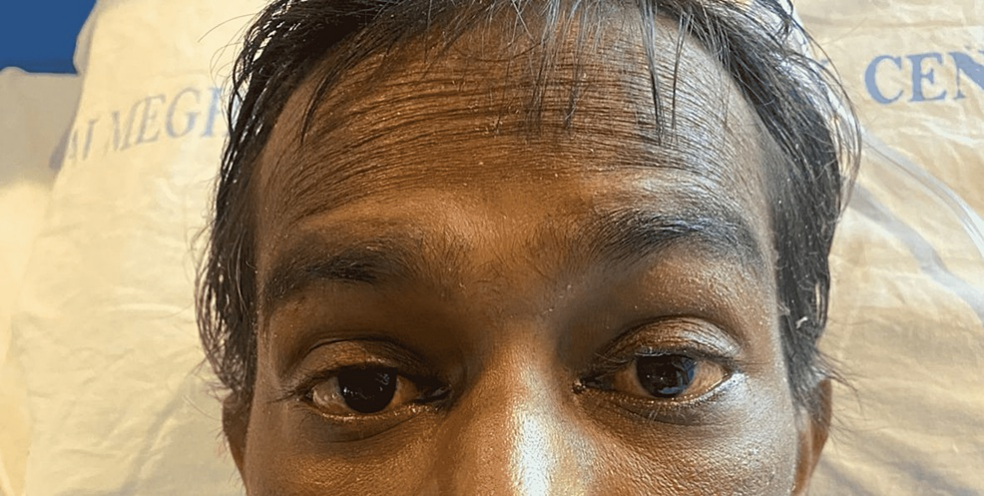
Yellowish discolouration of sclera

On general examination, pulse was 126 beats/min, blood pressure was 90/60 mm of hg, saturation 98% on room air, and deep icterus present (Figure 1). No signs of pedal edema, pallor, cyanosis, clubbing, or lymphadenopathy were seen. Systemic examination revealed the following: cardiovascular system - S1S2 heard, respiratory system - bilateral equal air entry present, per abdomen - soft and tender hepatomegaly. On central nervous system examination, he was semiconscious and disoriented. The rapid antigen test for coronavirus disease 2019 (COVID-19) was negative. Laboratory investigations were done and the results were suggestive of raised liver enzymes, hyperbilirubinemia, and coagulopathy (Table 1). Ultrasonography and computed tomography scans of the abdomen and pelvis were done, which were suggestive of hepatomegaly with grade I fatty liver.

**Table 1:** Laboratory findings

Complete blood count- parameters	
Total leukocyte count	8100 /dl
Haemoglobin	14.3 gm/dl
Mean corpuscular volume	84.6 fl
Platelet count	94,000 /dl
Prothrombin Time	32 seconds
International Normalized ratio	2.9
Kidney function test-	
Serum Urea	14 mg/dl
Serum Creatinine	0.8 mg/dl
Serum Sodium	139 mmol/l
Serum Potassium	5.4 mmol/l
Liver function test-	
Alanine Aminotransferase	952 units/l
Aspartate Aminotransferase	1343 units/l
Alkaline Phosphatase	161 gm/dl
Total Protein	9.4 gm/dl
Albumin	4.4 gm/dl
Globulin	5.0 mg/dl
Total Bilirubin	19.4 mg/dl
Bilirubin Conjugated	16.1mg/dl
Bilirubin Unconjugated	3.4 mg/dl
Serum Ammonia	64 mmol/l
Human immunodeficiency virus	Non Reactive
Hepatitis A, B, C	Non Reactive
Cytomegalovirus and Epstein-Barr virus	Non Reactive

Management

As the patient developed all the symptoms after consumption of sildenafil, suspicion of drug-induced acute fulminant hepatic failure was suspected. Tablet sildenafil was stopped. The patient was started on injectable antibiotics, glutathione 600 mg IV twice daily, Tablet Udiliv 300mg twice a day, and other supportive medication like a high carbohydrate diet through Ryles tube was given. The patient was transfused with five units of fresh frozen plasma during the hospital stay. Repeat laboratory tests were done, which showed improvement. The patient symptomatically improved and was discharged in stable condition.

## Discussion

Acute fulminant hepatic failure is a complex syndrome with multiple etiologies in which rapid deterioration of liver function occurs, resulting in sudden onset of jaundice, hepatic encephalopathy, and coagulopathy in absence of any history of liver disease [3]. Multiple etiologies resulting in acute fulminant hepatic failure such as idiopathic, drug toxicity such as acetaminophen, amiodarone, baclofen, isoniazid, ketoconazole, losartan, non-steroidal anti-inflammatory drugs (NSAIDS), statins, valproic acid, sulphonamides, amoxicillin-clavulanic acid, erythromycins, viral or autoimmune hepatitis, Wilson's disease of which viral or toxin-induced hepatitis are most common [4]. Zinc phosphide also can cause fulminant hepatitis [5]. Symptoms such as fever, malaise, loss of appetite, abdominal pain, jaundice, etc. lead to the development of encephalopathy. The patient presents with coagulopathy, which precedes the progression of hepatic encephalopathy to coma [6].

The cause of hepatic damage caused by sildenafil is unclear; however, it is degraded through the isoenzymes system (Cytochrome p450 3A4), and aberrant development of a hazardous by-product may underpin cases of liver toxicity. In certain cases, ischemia damage to the liver may be the cause of an immediate, self-limiting injury owing to hypotension or an abrupt cardiac event provoked by sildenafil-induced dilatation or sexual activity, especially when combined with nitrates [1].

According to a Dutch study, more than three-quarters of herbal medicines used to increase sexual potency include medicinal ingredient sildenafil or its analogues doses. However, up to now, just one report has been authored addressing liver damage induced by intake of tainted "Chinese herbal" supplement, “Tiger King", marketed for sexual purposes in a patient having a family history of cirrhosis, severe hepatitis, as well as moderate hepatitis on occasion [1].

## Conclusions

The most severe complication including mortality of drug-induced hepatotoxicity is acute liver failure. The clinical course was typical for a drug-induced liver injury and, in the absence of evidence for biliary disease and lack of other medication exposure, sildenafil is certainly suspect. There should be more awareness in routine patient care than in the regulatory and industry setting, especially in such fancy drugs. Immediate supportive management of the patient is required including liver transplantation if the clinical situation deteriorates.
